# Marginal notes, November 2024 – soil fever

**DOI:** 10.1099/jmm.0.001948

**Published:** 2024-12-13

**Authors:** T.J.J. Inglis

**Affiliations:** 1 PathWest Laboratory Medicine, Western Australian Country Health Service, Schools of Medicine and Biomedical Science, University of Western Australia, Perth, Australia

**Keywords:** *Burkholderia pseudomallei*, clinical microbiology, melioidosis, molecular biology, vaccines

The weather in Northern Australia at this time of year is a shock to those of us who work in more temperate climates. It is known as the Build Up and heralds the oncoming rainy season. Thus, it was in Darwin during the 2024 World Melioidosis Congress, a tightly focussed gathering of scientists and clinicians who share an interest in a potentially fatal but neglected tropical infection. In the longer-than-usual interval since the last melioidosis congress (Hanoi, 2019), detailed phylogeographic analysis of *Burkholderia pseudomallei* genomes shifted attention from the present-day melioidosis centre of gravity in Southeast Asia to a likely ancient origin in northern Australian [1]. It was, therefore, fitting that this recent insight from molecular microbiology on the origin of species should take place in a city named after Charles Darwin.

Regular contributors (‘melioidistas’) have a habit of coming up with distinctly new perspectives on persistent problems. A stand-out at this year’s congress was the account of lived experience with melioidosis from Sri Lanka [2]. A meticulous ethnographic survivor-driven approach told the story of personal and community disease impact and provided a model for community involvement in the co-design of future clinical research. Noting that bloodstream *B. pseudomallei* infection and sepsis are common in Sri Lanka, a medical anthropological approach could be broadly applied to sepsis and bloodstream infection. Also coming out of Sri Lanka is the term ‘soil fever’, used for patient communication in a dedicated melioidosis clinic.

The good news from well-equipped clinical centres is that melioidosis in-hospital mortality rates have fallen [3]. Some of that can be attributed to earlier recognition, combined with prompt initial treatment. The prolonged biphasic (initial and eradication) treatment recommendations are now under review, and preliminary data raise questions about how best to select patients for lengthy eradication therapy. While close follow-up may be possible in well-resourced health systems, high-prevalence settings in low- and middle-income countries have to tread a short-course therapy path with caution. Those who remember the early days of tuberculosis short-course therapy will be familiar with some of the practical considerations.

There is cause for cautious optimism about a future melioidosis vaccine. Several subunit vaccine candidates are in pre-clinical development, of which the most promising is a glycoconjugate based on *B. pseudomallei* capsular polysaccharide [4]. This has been a long and winding road since originally proposed. However, the current favourites have reached a point where the global reach of melioidosis makes a strong case for vaccine use and has implications for vaccine trial design.

One of the consistent features of the melioidosis congress has been the organisers' recognition of the historic roots of this body of work. Though senior participants may have heard of Whitmore and Krishnaswami’s discovery several times, the story gets better with each telling [5]. Some aspects of the early years of melioidosis have yet to be unearthed from dusty colonial archives. There is plenty to keep the melioidistas busy for years to come.

Conference abstracts can be found on the conference website [https://www.wmc2024.org.au/abstracts].

The next World Melioidosis Congress will be in Kuching, Malaysia.
